# Chronological age and failure to rescue after colorectal cancer surgery: A multicentre cohort study

**DOI:** 10.1007/s40520-026-03508-y

**Published:** 2026-09-07

**Authors:** Tomasz Cwaliński, Michał Kazanowski, Jacek Piątkowski, Maciej Ciesielski, Marcin Januszkiewicz, Zuzanna Bigus, Patryk Kaczor, Bartosz Kapturkiewicz, Paweł Lesiak, Sergii Girnyi, Damian Nowiński, Piotr Kurek, Marek Jackowski, Marek Bębenek, Mateusz Jagielski, Luigi Marano

**Affiliations:** 1 Department of General Surgery and Surgical Oncology, Saint Wojciech Hospital, Nicolaus Copernicus Health Center, Gdańsk, Poland; 2 Department of Oncology and Haematology, Faculty of Medicine, Wroclaw University of Science and Technology, Wroclaw, Poland; 3 First Department of Surgical Oncology, Lower Silesian Oncology, Pulmonology and Hematology Center, Wroclaw, Poland; 4 Department of General, Gastroenterological and Oncological Surgery, Collegium Medicum, Nicolaus Copernicus University in Toruń, Toruń, Poland; 5 Department of Oncological Surgery, Gdynia Oncology Centre, Gdynia, Poland; 6 Department of Oncological Surgery, Faculty of Health Sciences with the Institute of Maritime and Tropical Medicine, Medical University of Gdańsk, Gdańsk, Poland; 7 Department of Minimally Invasive and Oncological General Surgery, Voivodeship Hospital in Bialystok, Bialystok, Poland; 8 Department of Surgery, Dnipro State Medical University, Vernadskoho st 9, Dnipro, 49044 Ukraine

**Keywords:** Chronological age, Colorectal cancer, Failure to rescue, Geriatric assessment, Postoperative complications, Postoperative mortality

## Abstract

**Background:**

Whether advancing chronological age is associated more strongly with serious postoperative events or with survival after an event remains uncertain.

**Aims:**

To compare the association of chronological age with major postoperative events and failure to rescue after colorectal cancer surgery.

**Methods:**

We analyzed 1,124 adults treated at five Polish centers between January 2022 and August 2025. Major events were Clavien–Dindo ≥grade IIIa; failure to rescue was cumulative 90-day death after such an event. Chronological age was analysed per 10-year increase. Multivariable logistic regression examined major events and Firth regression examined failure to rescue.

**Results:**

Major events occurred in 145 patients; failure to rescue was evaluable in 134 and occurred in 24. Chronological age was not associated with major events (adjusted odds ratio per decade 1.09, 95% confidence interval 0.89–1.33) or nonfatal major morbidity. Failure-to-rescue odds increased per decade (odds ratio 2.02, 95% confidence interval 1.21–3.63); model-standardized probabilities were 11.1% at age 65 and 32.5% at age 85 years. Sensitivity analyses were directionally consistent, but the interval included no association when every model-eligible missing post-event outcome was assigned to death.

**Discussion:**

The chronological-age gradient was concentrated in survival after a serious event. Frailty and physiological reserve were not measured; reduced reserve remains a plausible explanation rather than a demonstrated mechanism.

**Conclusions:**

Chronological age may help identify patients who warrant intensified postoperative surveillance and early escalation, while formal geriatric and frailty assessment remains necessary to characterize biological vulnerability.

**Supplementary Information:**

The online version contains supplementary material available at https://doi.org/10.1007/s40520-026-03508-y.

## Introduction

Colorectal surgery is increasingly undertaken in older adults, making age-sensitive perioperative assessment a routine clinical problem [1]. Chronological age is convenient but only partly captures biological vulnerability; people of the same age may differ substantially in comorbidity, frailty, muscle strength, cognition, nutrition, and functional independence [2–6]. Prospective abdominal-surgery cohorts suggest that frailty and handgrip strength add clinically relevant information about early recovery beyond age alone [2–4]. A recent colorectal meta-analysis likewise associated frailty with major complications and postoperative mortality [5]. Age is therefore better regarded as one component of assessment than as a direct measure of physiological reserve [1–6].

Frailty describes vulnerability before a stressor and is distinct from comorbidity and disability [7]. Physiological reserve is the capacity to tolerate a perturbation without sustained dysfunction [8, 9], whereas physical resilience describes resistance to or recovery from functional decline after a stressor [9–11]. In surgery, outcome may reflect baseline reserve, operative stress, complications, and recovery [8–11]. These constructs are related but not interchangeable, and none can be inferred from chronological age alone.

Failure to rescue (FTR), usually defined as death after a postoperative complication, offers a clinically useful way to distinguish event occurrence from survival after the event [12]. The concept arose from the observation that differences in surgical mortality may reflect how hospitals manage serious complications more than how often those complications occur [12, 13]. Definitions nevertheless vary in their numerator, complication denominator, and mortality horizon [14]. In colorectal surgery, older age has been associated with a disproportionate increase in death after complications, including anastomotic leak, even when the gradient in complication frequency is less marked [15–19]. Recognition of deterioration, communication, access to critical care, and timely intervention are also part of the rescue pathway [13, 20, 21].

Using a multicentre Polish colorectal cancer cohort, we examined whether chronological age was more strongly associated with FTR than with major-event occurrence. Age was modelled continuously, with secondary age groups and model-standardised absolute probabilities. We hypothesised that the age association would be stronger for conditional mortality after a major event. The design assessed an age gradient in rescue, not frailty or reduced physiological reserve as mediators.

## Methods

### Study design and setting

This retrospective multicenter cohort study was a secondary analysis of the final Polish Analysis of Textbook Outcome in Colorectal Cancer Surgery (PolATOCCS) registry. The source registry included consecutive adult patients treated at five Polish tertiary referral centers between January 2022 and August 2025. Data collection followed the broader ATOCCS clinical framework [22].

The study protocol received a positive opinion from the Ethics Committee of the Academy of Applied Medical and Social Sciences, Elbląg, Poland (KB10/2024; 10 July 2024), covering the entire study period and all data included in the present analysis. The Ethics Committee resolution expressly permitted the retrospective inclusion and analysis of routinely collected clinical data from patients treated before the approval date, provided that no study-specific intervention or prospective research enrolment had occurred before approval. Data from patients treated after the approval date were collected within the same approved observational framework. Given the observational nature of the study and the use of pseudonymized routinely collected clinical data, the requirement for study-specific informed consent was waived. Written informed consent for surgical treatment was obtained in accordance with local institutional policies. All participating centers contributed pseudonymized data in accordance with institutional regulations and applicable data-protection requirements. The study was conducted in accordance with the Declaration of Helsinki. Analyses were performed within the study team’s controlled research environment, and reporting followed the Strengthening the Reporting of Observational Studies in Epidemiology (STROBE) recommendations for cohort studies [23].

### Participants

The full-registry analysis retained all 1,124 source records to describe the entire clinical spectrum entered by participating centers. The parent age-outcomes protocol specified age 18 years or older, ASA class I–III, no pathological stage IV disease, and colorectal resection. Twenty-four unique records did not meet at least one of these criteria (12 ASA IV–V, 11 stage IV, and two nonresection procedures; categories overlapped), leaving 1,100 patients for a sensitivity analysis. Missing eligibility fields were not interpreted as exclusions when a center had entered the patient in the registry cohort.

### Exposure and covariates

Chronological age was complete and was modelled primarily per 10-year increase. Age groups (< 75, 75–79, and ≥ 80 years) provided secondary clinical contrasts. Recorded covariates were sex, body mass index (BMI), ASA physical status, tumour site, pathological stage, neoadjuvant treatment, operative approach, combined-organ resection, study centre, and operative period. Converted procedures remained assigned to the intended laparoscopic or robotic platform.

The registry included the age-adjusted Charlson Comorbidity Index (aCCI) [24]. Because aCCI incorporates chronological age, it was summarised descriptively but was not entered unchanged into models that already contained age. Conventional age points were subtracted to derive an approximate comorbidity-only component for sensitivity analysis.

The registry was not designed as a geriatric assessment study. It did not collect a validated frailty instrument or baseline measures of activities of daily living, cognition, gait speed, handgrip strength, or exercise capacity. A validated frailty or physiological-reserve measure therefore could not be reconstructed retrospectively. ASA physical status, BMI, and aCCI were treated as conventional clinical variables and were not considered substitutes for frailty or physiological reserve.

### Outcomes and operational definitions

The primary outcome was FTR, defined as cumulative 90-day death among patients who experienced a major postoperative event [12, 14]. A major event was the highest recorded Clavien–Dindo grade ≥IIIa [25]. To separate severe morbidity among survivors from the mortality embedded in grade V, major morbidity excluding grade V (grades IIIa–IVb) was also examined.

Cumulative 90-day mortality was coded as positive when death appeared in either the 30- or 90-day mortality field or when the highest Clavien–Dindo grade was V. Successful rescue was survival through 90 days among patients with a major event and evaluable mortality.

Secondary outcomes were 30-day mortality, cumulative 90-day mortality, major morbidity excluding grade V, anastomotic leak, surgical reintervention, 30-day and cumulative 90-day readmission, postoperative length of stay (LOS), and LOS longer than 14 days. Leak was evaluated only when reconstruction and leak status were both documented. Surgical reintervention denoted operative management of a postoperative complication.

### Missing data

Outcome-specific evaluable denominators were reported, and missing outcomes were not imputed. Models used complete cases for their included variables. Mortality was unavailable for 179 patients overall and for 11 patients after a major event; these missing post-event outcomes arose in one centre. The primary FTR analysis used available outcomes. Sensitivity analyses examined how unavailable mortality could influence the association rather than classifying missing records as survivors.

### Sample size

All available registry records were analyzed; no post hoc power calculation was used for this secondary question. Precision was described with 95% confidence intervals. Because FTR deaths were sparse, model complexity was limited and bias-reduced estimation was used.

### Statistical analysis

Continuous variables are presented as median (interquartile range), and categorical variables as n/N (%). Age-group comparisons used the Kruskal–Wallis test for continuous variables and Pearson’s chi-square or Fisher’s exact test for categorical variables, as appropriate. These comparisons were descriptive. Tests were two-sided, *p* < 0.05 was used as a conventional threshold, and no multiplicity adjustment was applied.

The association between age and major-event occurrence was estimated with multivariable logistic regression adjusted a priori for sex, BMI per 5 kg/m², ASA class III or higher, rectal tumor site, neoadjuvant treatment, operative approach, center, and operative period. The same adjustment set was used for major morbidity excluding grade V. Sparse mortality and FTR endpoints were analyzed with Firth bias-reduced logistic regression and profile-likelihood confidence intervals [26]. To limit overfitting, the primary FTR model included age, ASA class III or higher, and operative period. A broader sensitivity model added sex, tumor site, neoadjuvant treatment, operative approach, and center. Secondary models replaced continuous age with the two older age groups, using < 75 years as reference.

Adjusted absolute FTR probabilities were obtained by standardizing the primary Firth model across age while retaining the observed ASA and operative-period distribution of the complete-case FTR risk set. Ninety-five per cent simulation intervals used 10,000 coefficient draws. An exploratory age-by-ASA interaction assessed whether the age odds ratio differed between ASA I–II and ASA class III or higher.

Sensitivity analyses used the explicit mortality fields alone; added the comorbidity-only Charlson component; omitted each centre in turn; and applied broader case-mix adjustment. Missing post-event mortality was bounded by assigning every model-eligible unavailable outcome first to survival and then to death; all 1,024 possible assignments across the ten model-eligible records were also fitted as an unweighted stress test. These exercises were not multiple imputation.

Statistical analyses were performed using Stata version 19.0 (StataCorp LLC, College Station, TX, USA). Firth estimation used a custom penalised-likelihood implementation with profile-likelihood intervals. All primary fits converged, and a deterministic rerun reproduced the archived aggregate outputs.

## Results

### Study population and outcome completeness

**Fig. 1:**
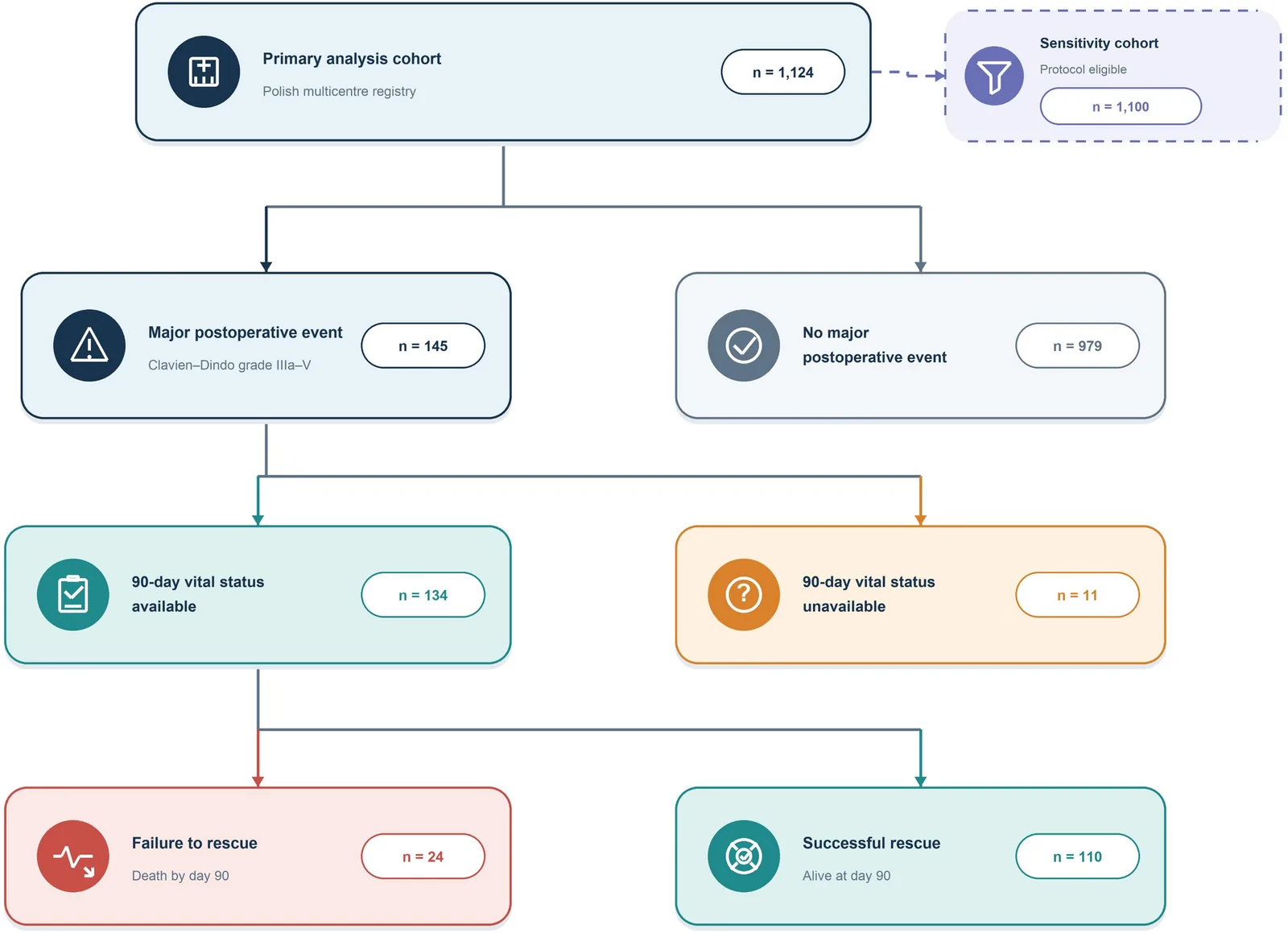
Cohort flow and formation of the failure-to-rescue risk set. The primary analysis included all 1,124 registry records. The protocol-eligibility sensitivity cohort excluded 24 unique records (12 ASA class IV–V, 11 pathological stage IV, and 2 nonresection procedures; categories overlapped), leaving 1,100 patients. Failure to rescue was evaluated among patients with a Clavien–Dindo grade IIIa–V event and known cumulative 90-day mortality; grade V was classified as death by day 90. ASA, American Society of Anesthesiologists

Cohort derivation, the protocol sensitivity populations, and formation of the FTR risk set are shown in Fig. 1. The age groups and baseline clinical characteristics are summarized in Table 1.

**Table 1:** Baseline characteristics according to age group

Characteristic	Overall	< 75	75–79	≥ 80	*p* value
Patients, n	1,124	766	210	148	—
Age, years	70 (64–76)	66 (60–71)	77 (76–78)	85 (82–87)	—
Male sex	625/1,124 (55.6)	443/766 (57.8)	101/210 (48.1)	81/148 (54.7)	0.041
BMI, kg/m²	26.6 (23.9–29.8)	27.0 (23.9–30.1)	26.8 (24.3–30.0)	25.2 (22.9–28.3)	0.001
ASA class ≥ III	347/1,106 (31.4)	171/753 (22.7)	89/206 (43.2)	87/147 (59.2)	< 0.001
Age-adjusted Charlson index	5 (4–6)	4 (4–5)	5 (5–6)	7 (6–8)	< 0.001
Approximate Charlson comorbidity component	2 (2–3)	2 (2–3)	2 (2–3)	3 (2–4)	< 0.001
Rectal cancer	436/1,123 (38.8)	339/766 (44.3)	68/210 (32.4)	29/147 (19.7)	< 0.001
Neoadjuvant treatment	267/1,078 (24.8)	219/736 (29.8)	32/199 (16.1)	16/143 (11.2)	< 0.001
Open approach	415/1,124 (36.9)	272/766 (35.5)	81/210 (38.6)	62/148 (41.9)	0.066
Laparoscopic approach	378/1,124 (33.6)	278/766 (36.3)	57/210 (27.1)	43/148 (29.1)	
Robotic approach	331/1,124 (29.4)	216/766 (28.2)	72/210 (34.3)	43/148 (29.1)	
Combined-organ resection	70/1,124 (6.2)	51/766 (6.7)	10/210 (4.8)	9/148 (6.1)	0.600
Pathological stage 0	66/1,124 (5.9)	53/766 (6.9)	8/210 (3.8)	5/148 (3.4)	0.202
Pathological stage I	317/1,124 (28.2)	218/766 (28.5)	57/210 (27.1)	42/148 (28.4)	
Pathological stage II	361/1,124 (32.1)	235/766 (30.7)	69/210 (32.9)	57/148 (38.5)	
Pathological stage III	369/1,124 (32.8)	255/766 (33.3)	72/210 (34.3)	42/148 (28.4)	
Pathological stage IV	11/1,124 (1.0)	5/766 (0.7)	4/210 (1.9)	2/148 (1.4)	

*Data are n/N (%) or median (IQR). Denominators vary because of missing data. Comparisons are descriptive; no p value is reported for age because age defines the groups. Global p values appear on the first row of multilevel variables. The final operative-period stratum contains 542 procedures from 2024 and 29 from 2025. ASA*,* American Society of Anesthesiologists; BMI*,* body mass index; IQR*,* interquartile range*

Mortality completeness varied by center; nearly all unavailable records arose from one site. Most patients with a major postoperative event had evaluable cumulative 90-day mortality. One patient in the FTR risk set lacked ASA class and was excluded from the primary adjusted model.

### Baseline clinical profile

Older patients more often had ASA class III or higher and greater recorded comorbidity. They also tended to have lower BMI, fewer rectal tumours, and less frequent neoadjuvant treatment. Operative approach and pathological stage were broadly similar across age groups (Table 1).

### Major events and rescue outcomes

Major postoperative events and mortality became more frequent across age strata in crude comparisons. The gradient was attenuated after grade V was excluded, indicating that fatal events contributed materially to the crude age difference in the composite major-event endpoint. Leak, reintervention, readmission, and postoperative LOS showed no clear age gradient (Table 2).

**Table 2:** Postoperative outcomes according to age group

Outcome	Overall	< 75	75–79	≥ 80	*p* value
Major postoperative event (Clavien–Dindo IIIa–V)	145/1,124 (12.9)	82/766 (10.7)	33/210 (15.7)	30/148 (20.3)	0.003
Major morbidity excluding grade V (IIIa–IVb)	121/1,124 (10.8)	76/766 (9.9)	24/210 (11.4)	21/148 (14.2)	0.291
30-day mortality	20/946 (2.1)	3/624 (0.5)	9/186 (4.8)	8/136 (5.9)	< 0.001
Cumulative 90-day mortality	25/945 (2.6)	6/624 (1.0)	9/185 (4.9)	10/136 (7.4)	< 0.001
Failure to rescue	24/134 (17.9)	6/75 (8.0)	9/30 (30.0)	9/29 (31.0)	0.003
Anastomotic leak	55/944 (5.8)	32/646 (5.0)	13/166 (7.8)	10/132 (7.6)	0.241
Surgical reintervention	103/1,124 (9.2)	62/766 (8.1)	22/210 (10.5)	19/148 (12.8)	0.143
30-day readmission	45/1,108 (4.1)	33/762 (4.3)	7/203 (3.4)	5/143 (3.5)	0.797
Cumulative 90-day readmission	110/1,106 (9.9)	84/760 (11.1)	15/203 (7.4)	11/143 (7.7)	0.189
Postoperative LOS, days	6.2 (5.0–8.1)	6.2 (5.0–8.0)	7.0 (5.0–9.0)	6.1 (5.0–9.0)	0.344
Postoperative LOS > 14 days	79/1,114 (7.1)	48/763 (6.3)	20/205 (9.8)	11/146 (7.5)	0.224

*Data are n/N (%) or median (IQR). Failure to rescue denotes cumulative 90-day death after a major event among patients with evaluable mortality. Cumulative 90-day mortality was positive when an explicit field recorded death or the highest grade was V. Other unavailable outcomes remained missing. IQR*,* interquartile range; LOS*,* length of stay*

### Adjusted association of age with failure to rescue

After case-mix adjustment, advancing age was not associated with major-event occurrence or with major morbidity excluding grade V. In contrast, age was associated with FTR and with both mortality endpoints (Table 3). Categorical age contrasts pointed in the same direction but were imprecise because deaths after a major event were few (Online Resource 1, Table S1).

**Table 3:** Adjusted associations of age with postoperative outcomes

Endpoint	Age contrast and model	*N*	Events	Adjusted OR (95% CI)	*p* value
Major postoperative event	Per 10 years; multivariable logistic	1,033	138	1.09 (0.89–1.33)	0.395
Major morbidity excluding grade V	Per 10 years; multivariable logistic	1,033	114	0.98 (0.80–1.20)	0.844
30-day mortality	Per 10 years; parsimonious Firth	934	20	2.89 (1.63–5.34)	< 0.001
Cumulative 90-day mortality	Per 10 years; parsimonious Firth	933	25	2.27 (1.39–3.83)	< 0.001
Failure to rescue	Per 10 years; primary Firth	133	24	2.02 (1.21–3.63)	0.006

*Major-event models adjusted for sex*,* BMI per 5 kg/m²*,* ASA class III or higher*,* rectal tumour site*,* neoadjuvant treatment*,* operative approach*,* centre*,* and operative period. Parsimonious Firth models adjusted for ASA class III or higher and operative period. Confidence intervals and p values for Firth models are profile-likelihood estimates. ASA*,* American Society of Anesthesiologists; BMI*,* body mass index; CI*,* confidence interval; OR*,* odds ratio*

**Fig. 2:**
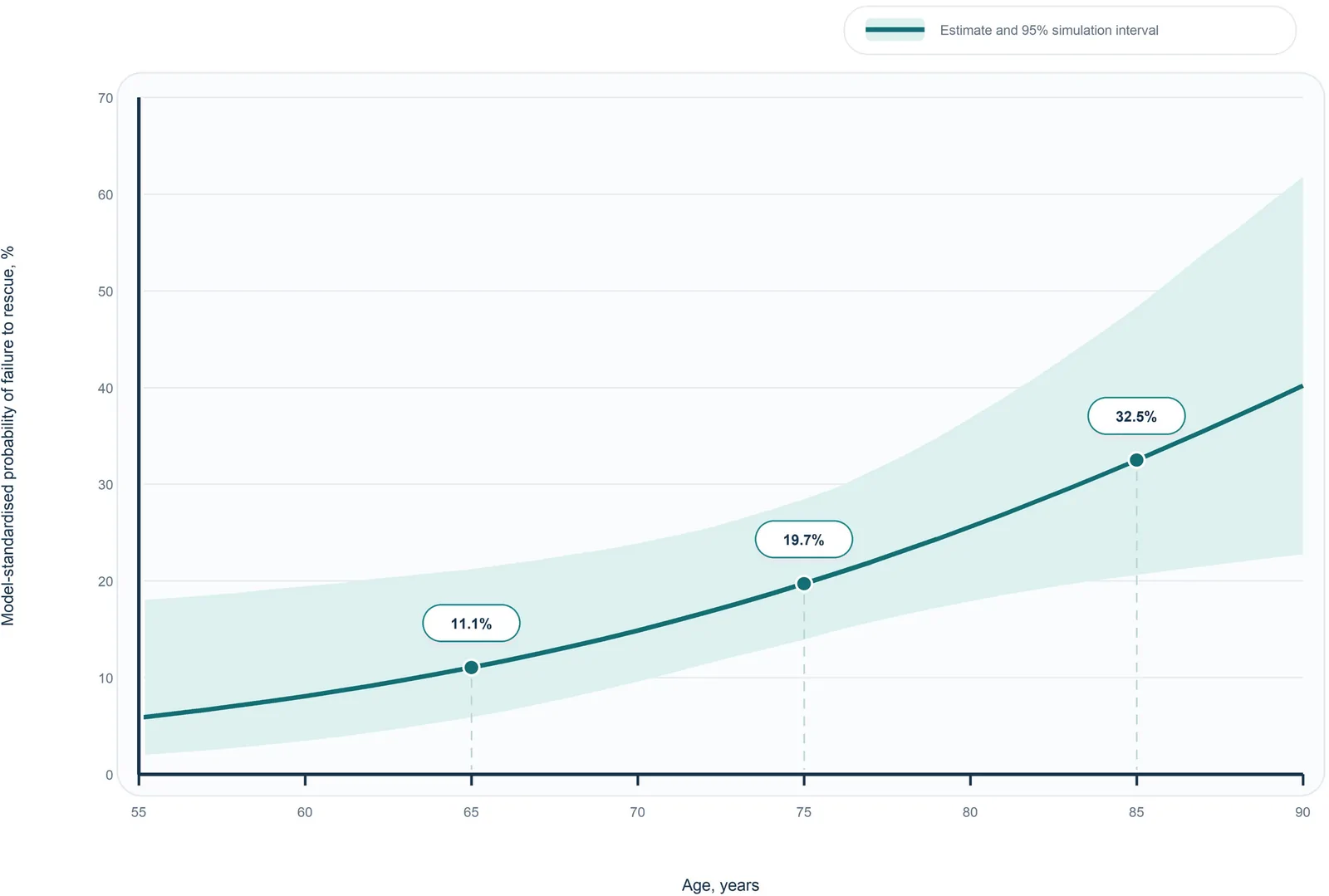
Model-standardised probability of failure to rescue according to chronological age. The solid line is the mean probability from the primary Firth model; shading denotes the 95% simulation interval from 10,000 coefficient draws. Standardisation retained ASA class and operative period at their observed distribution in the complete-case FTR risk set (*n* = 133; 24 deaths). Points identify ages 65, 75, and 85 years. The widening interval at older ages reflects increasing uncertainty. FTR, failure to rescue

Model standardization indicated a progressive rise in adjusted FTR probability across age (Fig. 2). Joint age–ASA profiles were consistent with greater conditional mortality among older patients with higher ASA class, although the exploratory interaction did not provide clear evidence that the age effect differed by ASA stratum (Online Resource 1, Table S2).

### Sensitivity analyses

The age–FTR estimate retained the same direction under broader adjustment, explicit mortality coding, restriction to the parent eligibility cohort, exclusion of individual centres, and addition of the derived comorbidity-only Charlson component; all corresponding point estimates remained above 1.0. The interval included no association only under the adverse scenario that assigned every model-eligible missing post-event outcome to death (Online Resource 1, Table S1). Conditional outcomes after a major event are presented descriptively in Online Resource 1, Table S3.

## Discussion

In this multicentre cohort, chronological age was more closely associated with survival after a major postoperative event than with occurrence of the event itself. Once case mix was considered, chronological age showed no independent association with major-event occurrence or nonfatal major morbidity, whereas the probability of FTR increased across age. The model-standardised probability rose from approximately one in nine at age 65 to almost one in three at age 85, making the clinical gradient more tangible than the odds ratio alone. Clinically, the signal points to the period after a complication: older patients who deteriorate may warrant particularly close surveillance and timely multidisciplinary escalation. This observation does not imply that chronological age measures frailty or physiological reserve, and age should not be used alone to guide treatment.

The pattern accords with earlier colorectal studies, although their definitions and follow-up intervals differ [14–19]. Nationwide German data showed that the oldest patients experienced disproportionate mortality once a postoperative complication occurred, even when the complication gradient was less pronounced [15]. A Spanish colon cancer cohort also identified age as a correlate of 90-day FTR [16]. Finnish population-based studies emphasized organ failure and timing of severe anastomotic leakage as determinants of rescue [17, 19], while a binational registry linked older age, high ASA class, and sepsis with death after leakage [18]. The present analysis adds continuous-age estimates and absolute model-standardized probabilities, but it does not establish a biological mechanism.

Chronological age is readily available but biologically imprecise [1–6]. Patients of the same age may differ considerably in frailty, muscle strength, nutrition, cognition, and functional independence [1–8]. Contemporary frameworks also distinguish baseline physiological reserve from the dynamic capacity to recover after stress [8–11]. Neither frailty, physiological reserve, resilience, nor recoverability was measured in this registry. Because the components required for a validated retrospective frailty measure were absent, we could not determine whether the age–FTR association would persist after adjustment for frailty. FTR should not be treated as a biomarker of any of these constructs. The association may reflect unmeasured biological vulnerability, event severity, treatment preferences, rescue processes, or a combination of these factors; reduced physiological reserve is therefore a plausible hypothesis rather than a demonstrated mediator.

Failure to rescue is not solely a patient characteristic; it also reflects how a clinical system recognizes and responds to deterioration [12, 13, 20, 21]. Access to imaging and critical care, communication across teams, timing of reintervention, staffing, and escalation decisions may all influence survival after a complication [20, 21]. The age association remained directionally similar when centers were omitted in turn. Even so, the registry did not capture deterioration-to-escalation intervals, critical-care capacity, or treatment-limitation decisions, so patient and system contributions cannot be separated.

These findings do not support withholding colorectal cancer surgery because of chronological age. A more useful implication is that an older patient who develops a major complication may merit a lower threshold for intensified monitoring and multidisciplinary escalation. Preoperative assessment should combine chronological age with formal geriatric assessment, validated frailty measurement, physical performance, nutrition, cognition, and comorbidity [1, 5, 6, 8]. Future prospective cohorts should collect these domains directly and pair them with time-resolved measures of deterioration and escalation. After surgery, structured surveillance and prompt review may be particularly relevant for patients with established vulnerability [21, 27]. Continuous ward monitoring may detect abnormalities earlier, although evidence that it improves patient-level outcomes remains incomplete [27]. The effectiveness of any rescue pathway cannot be inferred from the present observational data.

The analysis used consecutive records from five centers, considered age continuously, and separated event occurrence from conditional mortality. Bias-reduced regression limited small-sample bias in the sparse FTR models [26], and explicit denominators made outcome availability visible. The clinically important uncertainty lies in mortality ascertainment: 11 post-event outcomes were missing, all in one center. Estimates were stable under several sensitivity analyses, but the adverse missing-outcome scenario moved the confidence interval across the null. The primary finding should therefore be interpreted as a consistent association under observed and several plausible assumptions, not as evidence immune to informative missingness.

Other limitations also matter. The retrospective secondary design permits residual confounding and coding variation. The full-registry primary analysis included 24 records outside at least one parent age-outcomes eligibility criterion, although the restricted estimate was similar. FTR deaths were few, limiting model complexity and exploration of non-linearity or interaction. Highest Clavien–Dindo grade provided a common severity framework [25] but combined clinically different events. Cause of death, event timing, organ failure, functional recovery, treatment goals, and validated measures of frailty, baseline function, cognition, physical performance, or physiological reserve were unavailable; this precluded frailty-adjusted or mechanistic analyses. Generalizability beyond Polish tertiary centers is uncertain, and related analyses from the same cohort require transparent disclosure to avoid redundant publication.

## Conclusions

Older chronological age appeared to be more closely associated with survival after a major postoperative event than with event occurrence. The finding identifies an observable risk gradient but does not establish frailty or reduced physiological reserve as its mechanism. Chronological age may help identify patients who require particular attention to postoperative rescue, but it should neither substitute for formal geriatric and frailty assessment nor be used alone to determine operative eligibility. Prospective studies combining validated frailty and functional assessments with time-resolved measures of deterioration and escalation are needed to distinguish patient vulnerability from care-process effects.

## Supplementary Information

Below is the link to the electronic supplementary material.

Supplementary Material 1

## Data Availability

Individual-level data are not publicly available because they contain protected patient information and are subject to data-protection, ethics, and participating-center restrictions. A deidentified analytic dataset may be considered for controlled access. Requests should be directed to the corresponding author and would require approval from the relevant data controllers, participating centers, and ethics bodies.

## Declarations

### Related publication

A separate article based on the same Polish multicentre source cohort has been published [28]. That article examined component- and domain-specific Textbook Outcome failure according to operative approach, with particular attention to robotic surgery. The present study addresses the association of chronological age with major postoperative events and failure to rescue; its research question, primary exposure, endpoints, and analyses are distinct.
