# Supplementary material for: Chronological age and failure to rescue after colorectal cancer surgery: A multicentre cohort study
Source: Aging Clin Exp Res. 2026 Sep 7;38(1):205. doi: 10.1007/s40520-026-03508-y (PMC13653820; doi:10.1007/s40520-026-03508-y)
Supplement: Supplementary file 1 [file 40520_2026_3508_MOESM1_ESM.docx]

**Online Resource 1**

Chronological Age and Failure to Rescue After Colorectal Cancer Surgery: A Multicentre Cohort Study

Tomasz Cwaliński^1^, Michał Kazanowski^2,3^, Jacek Piątkowski^4^, Maciej Ciesielski^5,6^, Marcin Januszkiewicz^7^, Zuzanna Bigus^1^, Patryk Kaczor^4^, Bartosz Kapturkiewicz^2,3^, Paweł Lesiak^3^, Sergii Girnyi^1^, Damian Nowiński^7^, Piotr Kurek^5^, Marek Jackowski^4^, Marek Bębenek^2,3^, Mateusz Jagielski^4^, Luigi Marano^8*^

Corresponding author: Luigi Marano

**Supplementary Table S1 Sensitivity analyses for failure to rescue**

| Sensitivity analysis | Age contrast and model | N | Events | Adjusted OR (95% CI) | p value |
| --- | --- | --- | --- | --- | --- |
| Broader case-mix adjustment | Per 10 years; Firth | 130 | 24 | 1.81 (1.05–3.41) | 0.032 |
| Age 75–79 vs <75 | Categorical age; Firth | 133 | 24 | 4.00 (1.31–12.87) | 0.015 |
| Age ≥80 vs <75 | Categorical age; Firth | 133 | 24 | 3.37 (1.08–10.98) | 0.036 |
| Explicit mortality fields only | Per 10 years; Firth | 133 | 22 | 2.13 (1.25–3.96) | 0.004 |
| Parent eligibility cohort | Per 10 years; Firth | 127 | 21 | 2.11 (1.24–3.91) | 0.005 |
| Centre with greatest mortality missingness excluded | Per 10 years; Firth | 112 | 23 | 1.98 (1.19–3.59) | 0.007 |
| All model-eligible missing outcomes assigned survival | Per 10 years; Firth | 143 | 24 | 2.13 (1.27–3.83) | 0.003 |
| All model-eligible missing outcomes assigned death | Per 10 years; Firth | 143 | 34 | 1.39 (0.93–2.16) | 0.113 |

*All estimates concern failure to rescue. The primary Firth model adjusted for ASA class III or higher and operative period. The broader model additionally included sex, tumour site, neoadjuvant treatment, approach, and centre. 130 patients entered its complete-case FTR model. Extreme assignments applied only to the ten missing outcomes with complete primary-model covariates and are stress tests, not multiple imputation.*

**Supplementary Table S2 Joint age–ASA rescue profiles**

| Age stratum | ASA stratum | Major events | Mortality evaluable, n | Failure to rescue | Successful rescue |
| --- | --- | --- | --- | --- | --- |
| <75 | ASA I–II | 59 | 55 | 3/55 (5.5) | 52/55 (94.5) |
| <75 | ASA ≥III | 22 | 19 | 3/19 (15.8) | 16/19 (84.2) |
| ≥75 | ASA I–II | 28 | 28 | 6/28 (21.4) | 22/28 (78.6) |
| ≥75 | ASA ≥III | 34 | 31 | 12/31 (38.7) | 19/31 (61.3) |

*This table is descriptive. Two patients with a major event lacked ASA class; one also lacked evaluable cumulative 90-day mortality. The exploratory continuous age-by-ASA interaction did not show clear evidence of effect modification. ASA, American Society of Anesthesiologists.*

**Supplementary Table S3 Conditional outcomes after a major postoperative event**

| Conditional outcome | Overall | <75 | 75–79 | ≥80 |
| --- | --- | --- | --- | --- |
| Major postoperative events, n | 145 | 82 | 33 | 30 |
| Clavien–Dindo grade III | 103/145 (70.3) | 68/82 (82.9) | 18/33 (54.5) | 16/30 (53.3) |
| Clavien–Dindo grade IV | 19/145 (13.1) | 8/82 (9.8) | 6/33 (18.2) | 5/30 (16.7) |
| Clavien–Dindo grade V | 24/145 (16.6) | 6/82 (7.3) | 9/33 (27.3) | 9/30 (30.0) |
| Failure to rescue | 24/134 (17.9) | 6/75 (8.0) | 9/30 (30.0) | 9/29 (31.0) |
| Successful rescue | 110/134 (82.1) | 69/75 (92.0) | 21/30 (70.0) | 20/29 (69.0) |
| Reintervention | 102/145 (70.3) | 61/82 (74.4) | 22/33 (66.7) | 19/30 (63.3) |
| Death after reintervention | 12/95 (12.6) | 4/55 (7.3) | 6/22 (27.3) | 2/18 (11.1) |
| Death without reintervention | 12/39 (30.8) | 2/20 (10.0) | 3/8 (37.5) | 7/11 (63.6) |
| Leak among evaluable major events | 51/125 (40.8) | 30/71 (42.3) | 12/26 (46.2) | 9/28 (32.1) |
| Death after leak | 6/49 (12.2) | 1/28 (3.6) | 4/12 (33.3) | 1/9 (11.1) |

*Data are n/N (%), unless stated otherwise. Failure-to-rescue and successful-rescue denominators include only patients with evaluable cumulative 90-day mortality. Event timing, treatment indications, escalation intervals, and treatment-limitation decisions were unavailable; these profiles are descriptive and do not estimate treatment effects.*
